# SKIN-BAR project: protocol for a prospective cohort study investigating skin barrier function and pressure injury development in critically ill adults

**DOI:** 10.1136/bmjopen-2026-124660

**Published:** 2026-09-21

**Authors:** Tuğba Erdem, Meryem Yıldız Ayvaz, Sena Şükran Gura, Mehmet Eren Açık, Evren Şentürk

**Affiliations:** 1 School of Nursing, Koç University, Istanbul, Turkey; 2 Department of Anesthesiology and Reanimation Intensive Care Unit, Koç University Hospital, Istanbul, Turkey; 3 Department of Anesthesiology and Intensive Care Medicine, School of Medicine, Koç University, Istanbul, Turkey

**Keywords:** Intensive Care Units, Nursing Care, DERMATOLOGY, Adult intensive & critical care

## Abstract

**Introduction:**

Pressure injuries (PIs) remain a common and costly complication among critically ill patients despite advances in prevention strategies. Current assessment methods rely primarily on visual skin inspection and risk assessment tools, which may fail to detect early physiological alterations preceding visible tissue damage. Biophysical parameters, including transepidermal water loss, stratum corneum hydration, skin pH and skin temperature, have emerged as promising indicators of skin barrier dysfunction; however, longitudinal evidence in intensive care populations remains limited. This study aims to investigate temporal changes in skin barrier function in pressure injury-prone and affected skin and to identify clinical and physiological factors associated with skin barrier dysfunction and pressure injury development.

**Methods and analysis:**

The SKIN-BAR project is a prospective observational cohort study to be conducted in an adult intensive care unit in Turkey. A total of 165 critically ill adults will be recruited and followed throughout their intensive care unit (ICU) stay. Skin barrier function will be assessed using non-invasive measurements of transepidermal water loss, stratum corneum hydration, skin pH and skin temperature. Measurements will be obtained within the first 4 hours of ICU admission, at 24, 48, 72 and 96 hours, on day 14, and weekly thereafter until ICU discharge. Clinical, laboratory, treatment-related and nursing care variables will also be collected. Linear mixed-effects models will be used to examine longitudinal changes in skin barrier function, while discrete-time survival analysis will be used to evaluate factors associated with incident pressure injury development.

**Ethics and dissemination:**

Ethical approval has been obtained from the Institutional Research Ethics Committee. Written informed consent will be obtained from participants or their legally authorised representatives before enrolment. Findings will be disseminated through publication in peer-reviewed journals, presentations at national and international scientific conferences and other academic dissemination activities.

**Trial registration number:**

NCT07560683.

**STRENGTHS AND LIMITATIONS OF THIS STUDY**

- This prospective observational cohort study will provide repeated longitudinal measurements of multiple objective skin barrier function parameters, including transepidermal water loss, stratum corneum hydration, skin pH and skin temperature throughout the intensive care stay.
- Standardised non-invasive biophysical measurements combined with comprehensive clinical, laboratory and nursing data will enable detailed evaluation of factors associated with skin barrier dysfunction and pressure injury development.
- The longitudinal design allows assessment of temporal changes in skin barrier function before and after pressure injury development, which has been insufficiently explored in previous studies.
- As a single-centre study conducted in one adult intensive care unit, the findings may have limited generalisability to other healthcare settings and patient populations.
- The observational design allows investigation of associations but does not permit causal inference regarding skin barrier alterations and pressure injury development.

## Introduction

 Pressure injuries (PIs) are localised tissue damages that typically develop over bony prominences as a result of prolonged pressure or the combined effect of pressure and shear forces on the skin and/or underlying soft tissue.^1^Patients monitored in intensive care unit (ICU) constitute a particularly high-risk group for PIs due to factors such as immobility, hypoperfusion, vasopressor use, inadequate nutrition, mechanical ventilation, invasive devices, fluid and electrolyte imbalances and haemodynamic instability, all of which reduce tissue tolerance and accelerate PI development.^2^

The prevalence and incidence of PIs acquired in intensive care settings remain substantial worldwide. In the DecubICUs study, PIs were identified in 6747 of 13 254 patients followed in 1117 ICUs, with 59.2% of these lesions developing during ICU stay.^3^ In addition to their clinical consequences, PIs impose a considerable economic burden on healthcare systems.^4–7^

The prevention and early detection of PIs are recognised as key indicators of nursing care quality. Current clinical guidelines recommend evidence-based interventions including risk assessment, comprehensive skin evaluation, protective skin care, regular repositioning, early mobilisation, nutritional support and the use of pressure-redistributing support surfaces.^1 8^ Among these strategies, skin assessment plays a central role in identifying early tissue damage and guiding preventive interventions.^9^ However, visual skin assessment remains dependent on clinician judgement and may be subject to variability, potentially limiting the objectivity and sensitivity of findings.^10^

Consequently, increasing attention has been directed toward objective approaches for assessing skin barrier function. Biophysical measurements obtained through biosensory technologies suggest that PI pathophysiology, including ischaemia, reperfusion injury, cellular death, inflammation and impaired lymphatic drainage, may be reflected by alterations in skin barrier integrity before visible clinical signs emerge.^11 12^ Parameters commonly associated with skin barrier function include transepidermal water loss (TEWL), skin pH, subepidermal moisture, stratum corneum hydration (SCH), erythema, skin temperature, sebum and transcutaneous oxygen levels.^13–16^

Although previous studies suggest that changes in biophysical parameters may indicate early impairment of the skin barrier, existing evidence remains fragmented. Most studies have focused on single parameters, included limited sample sizes, evaluated a restricted number of anatomical sites or lacked repeated measurements, particularly in critically ill populations.^17–19^ As a result, the longitudinal relationship between skin barrier function and PI development remains insufficiently understood.

To address these gaps, the SKIN-BAR project was designed to investigate skin barrier function in PI-prone and affected anatomical sites among ICU patients using repeated, non-invasive biosensor-based measurements. By integrating multiple biophysical parameters with detailed clinical data, this study aims to improve understanding of skin barrier alterations associated with PI development and to support the future identification of objective physiological markers for early PI detection and prevention.

### Objectives

This study aims to investigate skin barrier function in PI-prone and affected anatomical sites among ICU patients using non-invasive assessments of TEWL, skin pH, stratum corneum hydration and local skin temperature. Specifically, the study will evaluate longitudinal changes in skin barrier parameters, compare patients with and without PIs, examine relationships between biophysical measurements, identify clinical and physiological determinants of skin barrier dysfunction and explore differences according to PI stage and anatomical location.

## Methods and analysis

### Study design and setting

This study is designed as a prospective longitudinal cohort study conducted in an intensive care setting. The research will be carried out in the adult ICU of Koç University Hospital, Department of Anesthesiology and Reanimation in Turkey. The study is planned over a total period of 24 months, commencing in September 2026 and concluding in September 2028.

### Participants and sampling

All patients admitted to the ICU during the study period who meet the eligibility criteria will be invited to participate. Eligible patients who provide informed consent, either directly or via a legal representative, will be consecutively enrolled and followed longitudinally throughout their ICU stay.

The sample size was calculated using G*Power software based on previously reported differences in sacral stratum corneum hydration between patients who developed PIs and those who did not. In the reference study, sacral stratum corneum hydration was 17.7±3.78 AU in patients who developed pressure injuries and 20.0±3.92 AU in those who did not, corresponding to an effect size of approximately Cohen’s d=0.59.^20^ Assuming a two-sided significance level of 0.05, 90% statistical power and an allocation ratio of 1:2 based on the expected distribution of patients with and without pressure injury in the study setting, the minimum required sample size was calculated as 138 participants (46 with PI and 92 without PI). To account for an anticipated attrition rate of approximately 20%, the target sample size was increased to 165 participants.

### Eligibility criteria

Participants will be included if they are aged 18 years or older, undergo initial assessment within the first 4 hours of ICU admission, are haemodynamically stable to tolerate repeated measurements and have intact skin integrity at ICU admission. Written informed consent will be obtained from all participants or their legally authorised representatives.

Patients will be excluded if they are unable to tolerate position changes, are in the post-cardiopulmonary resuscitation period at enrolment, experience cardiac arrest requiring resuscitation during follow-up, have severe oedema or subcutaneous fluid accumulation, present with pre-existing skin lesions prior to ICU admission or have systemic dermatological conditions that may directly affect skin barrier function.

### Recruitment

Consecutive patients admitted to the adult ICU will be screened daily for eligibility by the principal investigator. Eligible patients or their legally authorised representatives will receive verbal and written information about the study. Written informed consent will be obtained before any study-specific procedures are initiated.

### Data collection

Data collection will be conducted over the first 18 months of the study and will consist of two complementary components: (1) collection of sociodemographic and clinical data and (2) assessment of skin barrier function using non-invasive biosensor-based measurements. This approach is intended to provide a comprehensive evaluation of both physiological and clinical determinants of skin barrier integrity in critically ill patients.

### Baseline data and clinical assessments

Sociodemographic and clinical data will be collected using a structured data collection form developed by the research team. The form consists of 41 items and includes information regarding age, sex, primary medical diagnosis, reason for ICU admission, severity of illness, dependency status, history of skin and tissue damage, pharmacological treatments, respiratory and nutritional support, laboratory findings relevant to skin barrier function, selected arterial blood gas parameters and nursing care practices related to skin integrity (table 1).

**Table 1:** Outcome measures and assessment schedule

Outcome measure	Baseline (0–4 hours)	24 hours	48 hours	72 hours	96 hours	Day 14	Weekly until ICU discharge
Demographic characteristics	X	–	–	–	–	–	–
Clinical characteristics	X	X	X	X	X	X	X
APACHE II Score	X	–	–	–	–	–	–
Glasgow Coma Scale	X	X	X	X	X	X	X
Jackson/Cubbin Scale	X	X	X	X	X	X	X
mNutric Scale	X	–	–	–	–	–	–
Laboratory parameters	X	X	X	X	X	X	X
Nursing care variables	X	X	X	X	X	X	X
Transepidermal water loss (TEWL)	X	X	X	X	X	X	X
Stratum corneum hydration (SCH)	X	X	X	X	X	X	X
Skin pH	X	X	X	X	X	X	X
Local skin temperature	X	X	X	X	X	X	X
Pressure injury development	–	X	X	X	X	X	X
Pressure injury stage	–	X	X	X	X	X	X
Bates-Jensen wound assessment tool*	–	X	X	X	X	X	X
ICU length of stay	–	–	–	–	–	–	X†
ICU mortality	–	–	–	–	–	–	X†

* Assessed only in patients who develop pressure injuries.

† Recorded at ICU discharge or death.

APACHE II, Acute Physiology and Chronic Health Evaluation II; ICU, intensive care unit; mNutric, Modified Nutrition Risk in Critically Ill.

Baseline data will be collected within the first 4 hours following ICU admission. Relevant clinical variables will subsequently be recorded prospectively throughout the ICU stay to capture time-dependent changes and potential influencing factors.

Severity of illness will be assessed using the Acute Physiology and Chronic Health Evaluation II score, a validated instrument widely used to estimate disease severity and mortality risk among critically ill adults.^21^ Level of consciousness will be evaluated using the Glasgow Coma Scale and nutritional risk will be assessed using the Modified Nutrition Risk in Critically Ill score.^22 23^

PI risk will be assessed using the Jackson/Cubbin Pressure Area Risk Assessment Scale, which was specifically developed for intensive care populations and consists of 12 items evaluating age, tissue viability, skin condition, medical history, mobility, mental status, haemodynamic status, oxygen requirements, respiratory status, nutritional status, hygiene and continence. A total score of 29 or below is considered indicative of high PI risk.^24^

PIs will be classified according to the National PI Advisory Panel (NPIAP), European Pressure Ulcer Advisory Panel (EPUAP) and Pan Pacific PI Alliance (PPPIA) classification system.^1^

For participants who develop PIs, wound severity and healing progression will be evaluated using the Bates-Jensen Wound Assessment Tool (BWAT). The BWAT is a validated multidimensional instrument consisting of 13 wound characteristics that provide a standardised assessment of wound status over time.^25^

### Assessment of skin barrier function

Skin barrier function will be evaluated using a standardised measurement protocol and a dedicated Skin Integrity Monitoring Form developed by the research team. This form will be used to systematically record repeated measurements of TEWL, SCH, skin pH and local skin temperature across predefined anatomical regions.

### Measurement devices

Skin barrier function parameters will be assessed using non-invasive biophysical measurement devices. All devices are certified, used in clinical and academic research, and do not compromise skin integrity or patient comfort (table 2).

**Table 2:** Standardised measurement procedures for skin barrier function assessments

Parameter	Device	Anatomical sites	Measurement procedure	Unit
Transepidermal water loss (TEWL)	Vapometer (Delfin Technologies)	Sacrum, right and left trochanters, right and left scapular regions, right and left lateral lower extremities, posterior neck, corresponding control sites and PI-affected skin	Probe positioned perpendicular to the skin surface according to manufacturer recommendations	g/m²/h
Stratum corneum hydration (SCH)	MoistureMeterSC (Delfin Technologies)	Same sites as above	Probe applied using standardised contact pressure according to manufacturer recommendations	Arbitrary Units (AU)
Skin pH	PH5F pocket pH Meter (Apera Instruments)	Same sites as above	Flat electrode gently applied to the skin until a stable reading is achieved	pH units
Local skin temperature	Integrated temperature sensor of the Vapometer	Same sites as above	Recorded simultaneously during TEWL assessment	°C

Standardisation procedures.

✓ Measurements performed by trained researchers using a standardised protocol.

✓ Devices calibrated according to manufacturer recommendations before data collection sessions.

✓ Non-invasive assessment procedures used for all measurements.

✓ Identical measurement procedures applied across all anatomical sites and time points.

✓ Assessments performed within the first 4 hours of ICU admission, at 24, 48, 72 and 96 hours, on day 14, and weekly thereafter until ICU discharge.

✓ Corresponding control sites assessed alongside all PI-prone and affected anatomical sites.

✓ TEWL values calculated as the mean of two consecutive measurements.

✓ Measurements recorded immediately in the study database following assessment.

✓ Device probes cleaned and disinfected between patients using hospital-approved disinfectants in accordance with institutional infection prevention policies and manufacturer recommendations.

✓ Pressure injury sites assessed using the same standardised procedures, with measurements obtained from peri-wound skin when direct measurement of the wound bed is not appropriate.

PI, Pressure Injury.

### Transepidermal water loss and skin temperature

TEWL and local skin temperature will be measured using the Vapometer,^26^ a portable closed-chamber evaporimeter widely used for skin barrier function assessment. The device provides objective, non-invasive measurements of water vapour evaporation from the skin surface. To improve measurement reliability, two consecutive measurements will be obtained from each anatomical site, and the mean value will be used for analysis.^19 27^ The device will be calibrated according to the manufacturer’s recommendations before each measurement session.

### Stratum corneum hydration

SCH will be assessed using the MoistureMeterSC.^28^ This device uses dielectric measurement technology to quantify tissue water content and provide objective information regarding skin hydration status.^14 29^ The device has been widely used in skin integrity research and enables rapid, non-invasive assessment of tissue hydration.^30 31^

### Skin surface pH

Skin surface pH will be measured using the PH5F pH Meter.^32^ This portable, non-invasive device measures skin surface acidity through direct contact with the skin. Measurements will be continued until the reading stabilises, and calibration procedures will be performed according to the manufacturer’s instructions before each data collection session.

### Outcomes

#### Primary outcomes

The primary outcomes of the study are skin barrier function parameters measured at predefined anatomical sites, including TEWL, SCH, skin surface pH and local skin temperature. These parameters will be assessed repeatedly throughout the ICU stay to evaluate temporal changes in skin barrier function and to investigate their relationship with PI development.

#### Secondary outcomes

Secondary outcomes include PI incidence, time to PI development and PI severity according to the NPIAP, EPUAP and PPPIA classification system. Among patients who develop PIs, wound progression and healing status will be evaluated using the Bates-Jensen Wound Assessment Tool. Additional secondary outcomes include ICU length of stay and ICU mortality.

#### Exploratory outcomes

Exploratory analyses will investigate differences in skin barrier function parameters between patients who develop PIs and those who do not, associations among the measured biophysical parameters, longitudinal trajectories of skin barrier function across different anatomical sites and clinical and physiological determinants associated with alterations in skin barrier function.

#### Data collection methods

Biophysical measurements will be obtained from anatomical regions commonly identified as PI-prone sites in critically ill patients, including the sacrum, bilateral trochanters, bilateral lateral lower extremities, bilateral scapular regions and the posterior neck.

A repeated-measures approach will be used to evaluate both short-term and longitudinal changes in skin barrier function. This approach was selected based on previous studies in which repeated biophysical assessments were successfully applied to monitor temporal changes in skin integrity over time.^18 33–35^ The initial assessment will be performed within the first 4 hours of ICU admission. Subsequent measurements will be conducted at 24, 48, 72 and 96 hours, on day 14, and weekly thereafter until ICU discharge (table 2).

For each anatomical measurement site, a corresponding control area located approximately 10 cm away will also be assessed. To ensure consistency across repeated measurements, predefined circular measurement areas will be marked and used throughout follow-up.

All measurements will be conducted by trained researchers according to a standardised protocol. During each measurement session, one researcher will perform the measurements, a second researcher will verify the readings and a third researcher will record the data electronically. Paper-based forms will be available as a backup in case of technical issues.

If a PI develops, measurement procedures will be adapted according to injury stage. For Stage I PIs, measurements will be obtained directly from the affected site. For Stage II or more advanced PIs, measurements will be performed on the nearest adjacent area of intact skin. In both situations, the corresponding control site will also be assessed. Wound progression will be monitored using the Bates-Jensen Wound Assessment Tool.

To minimise the influence of mechanical loading, measurements will be performed following routine patient repositioning and approximately 15–20 min after pressure removal. All anatomical regions, except the sacrum, will be assessed bilaterally.

The duration of each measurement is approximately 15–20 s for most devices, whereas pH measurements require approximately 30 s for stabilisation. If haemodynamic instability occurs during the procedure, measurements will be immediately discontinued to ensure patient safety.

#### Data management

Each participant will be assigned a unique study identification number to ensure confidentiality. Personal identifiers will be removed from the analytical dataset and stored separately from study data in password-protected files accessible only to the principal investigator.

Data will be recorded using standardised case report forms and subsequently entered into a secure electronic database. Double-checking procedures will be performed regularly to verify data accuracy and completeness. Any inconsistencies identified during data entry will be reviewed against the original source documents and corrected where necessary.

All electronic data will be stored on password-protected institutional computers with regular backup. Paper-based study documents, including informed consent forms and source data, will be stored in locked cabinets within the research institution. Study records will be retained in accordance with institutional policies and applicable data protection regulations.

Only authorised members of the research team will have access to the study database. Data handling and storage will comply with national regulations and the principles of Good Clinical Practice.

#### Statistical analysis

Statistical analyses will be performed using IBM SPSS Statistics V.29.0 and, where required for time-to-event analyses, R statistical software.

Descriptive statistics will be used to summarise demographic, clinical and skin barrier function characteristics. Prior to inferential analyses, data will be examined for completeness, outliers and distributional assumptions. Continuous variables will be assessed for normality using visual inspection methods (histograms and Q–Q plots) and the Shapiro-Wilk test. Descriptive statistics will be reported as mean and SD or median and IQR, as appropriate. Continuous variables will be reported as mean and SD or median and IQR, as appropriate, whereas categorical variables will be presented as frequencies and percentages.

Changes in TEWL, SCH, skin pH and local skin temperature over time will be examined using repeated-measures analyses. To account for repeated observations within individuals and varying follow-up durations, linear mixed-effects models will be used as the primary analytical approach.

Comparisons between patients who develop PIs and those who do not will be performed using appropriate parametric or non-parametric methods according to data distribution. For patients who develop PIs, measurements obtained from affected sites will be compared with corresponding control sites using paired analyses.

The association between longitudinal skin barrier function parameters and incident PI development will be evaluated using discrete-time survival analysis. Follow-up will be divided according to the predefined skin assessment intervals, thereby accounting for the interval-censored nature of PI onset. TEWL, SCH, skin pH and local skin temperature will be incorporated as time-varying covariates using the most recent measurement available for each assessment interval. Death and ICU discharge before PI development will be considered competing events and will be explicitly accounted for in the time-to-event analyses. Potential determinants, including demographic characteristics, illness severity, PI risk scores, laboratory findings, treatments and nursing care practices, will first be examined in univariable analyses. Multivariable models will subsequently be constructed using a limited number of prespecified clinically relevant covariates, according to the number of observed PI events, to minimise model overfitting.

Inter-rater agreement for PI risk assessments and PI classification will be evaluated using Cohen’s kappa coefficient. In addition to statistical significance, effect sizes will be reported to facilitate interpretation of the clinical relevance of findings. Statistical significance will be set at p<0.05.

#### Patient and public involvement

Patients and members of the public were not involved in the design, conduct, reporting or dissemination plans of this research. The research question, study design, outcome measures and data collection procedures were developed by the research team based on existing evidence, clinical experience and identified gaps in the literature. The results of the study will be disseminated through peer-reviewed publications and scientific conferences to inform future clinical practice and research.

#### Ethics

Ethical approval for this study was obtained from the Biomedical Research Ethics Committee of Koç University (approval no: 2026.119.IRB2.059; date: 12 March 2026). Institutional permission to conduct the study was granted by Koç University Hospital. The study was registered at ClinicalTrials.gov (Identifier: NCT07560683).

Written informed consent will be obtained from all participants or their legally authorised representatives before enrolment. All procedures will be conducted in accordance with the principles of the Declaration of Helsinki.

#### Study status

At the time of manuscript submission, ethical approval, institutional permissions and trial registration had been completed. Participant recruitment had not yet commenced. Data collection is scheduled to begin in September 2026.

## Discussion

Growing evidence suggests that alterations in skin barrier function may occur before visible signs of PI become clinically apparent. Consequently, objective biophysical assessments have attracted increasing attention as potential tools for early detection of tissue damage and PI risk stratification. Parameters such as TEWL, SCH, skin pH and local skin temperature have all been proposed as indicators of skin barrier integrity; however, the available evidence remains limited and inconsistent.

Previous studies have demonstrated associations between abnormal biophysical parameters and PI development. In a cohort study conducted in the UK involving older adults with Stage I PIs, Abiakam *et al* reported substantially elevated TEWL values in PI sites compared with intact skin.^18^ Similarly, previous randomised controlled trial conducted in intensive care patients in Turkey demonstrated higher sacral TEWL values among patients who developed PIs, further supporting the potential relationship between skin barrier disruption and PI occurrence.^36^ Experimental and review studies have also suggested that increased TEWL may reflect early impairment of the stratum corneum and mechanical loading-related tissue damage.^37 38^

Evidence regarding other biophysical parameters remains less clear. He *et al* reported higher skin pH values and lower moisture levels in sacral and hip regions among critically ill patients with PIs, whereas Fischer *et al* demonstrated associations between skin pH and systemic inflammatory conditions such as sepsis and systemic inflammatory response syndrome.^20 33^ In contrast, Yılmaz and Günes found no significant differences in sacral skin temperature between patients who developed PIs and those who did not, although they highlighted the potential clinical importance of temperature fluctuations following repositioning.^39^ These findings suggest that individual parameters may capture different aspects of skin physiology and PI pathogenesis, reinforcing the need for comprehensive multidimensional assessment.

A further limitation of the existing literature is that most studies have evaluated only one or two skin barrier parameters at a time. For example, previous investigations have focused exclusively on sub-epidermal moisture,^40^ TEWL and pH,^33 36^ temperature^39^ or moisture, sebum and pH.^20^ Consequently, little is known about the interactions between different skin barrier parameters and their combined contribution to PI development. Existing studies are characterised by relatively small sample sizes, limited anatomical assessment sites and a lack of longitudinal measurements across the entire period of PI risk. Moreover, evidence from critically ill populations remains scarce.

The SKIN-BAR project was designed to address important gaps in the existing literature. By simultaneously evaluating TEWL, SCH, skin pH and local skin temperature across multiple PI-prone and affected anatomical sites, the project aims to provide a comprehensive understanding of skin barrier function in critically ill adults. Through repeated measurements and detailed clinical data collection, the study will investigate factors associated with skin barrier dysfunction and PI development.

As interest in objective skin assessment technologies continues to grow, robust longitudinal data are needed to support their implementation in clinical practice. The SKIN-BAR project aims to contribute to this evidence base and advance understanding of the physiological changes associated with PI development.

### Ethics and dissemination

#### Ethics approval and consent to participate

This study was approved by the Biomedical Research Ethics Committee of Koç University (approval no: 2026.119.IRB2.059; date: 12 March 2026). Institutional permission to conduct the study was obtained from Koç University Hospital. Written informed consent will be obtained from all participants or, where applicable, from their legally authorised representatives before enrolment.

As all study procedures involve non-invasive skin assessments using clinically approved devices, no additional risks beyond routine clinical care are anticipated. If any participant experiences clinical instability during data collection, study procedures will be discontinued immediately, and routine clinical care will take priority.

### Dissemination

The findings of this study will be disseminated through publication in peer-reviewed scientific journals and presentations at national and international scientific conferences. Study results may also contribute to future evidence syntheses, clinical practice recommendations and the development of objective approaches for monitoring skin barrier function and preventing pressure injuries in critically ill adults.
