# Supplementary material for: SKIN-BAR project: protocol for a prospective cohort study investigating skin barrier function and pressure injury development in critically ill adults
Source: BMJ Open. 2026 Sep 21;16(9):e124660. doi: 10.1136/bmjopen-2026-124660 (PMC13599962; doi:10.1136/bmjopen-2026-124660)
Supplement: Supplementary file 1 [file bmjopen-2026-124660.reviewer_comments.pdf]

PEER REVIEW HISTORY

BMJ Open publishes all reviews undertaken for accepted manuscripts. Reviewers are asked to complete a checklist review form and are provided with free text boxes to elaborate on their assessment. These free text comments are reproduced below.

ARTICLE DETAILS

Title (Provisional)

The SKIN-BAR Project: Protocol for a Prospective Cohort Study Investigating Skin Barrier Function and Pressure Injury Development in Critically Ill Adults

Authors

ERDEM, TUĞBA; YILDIZ AYVAZ, MERYEM; GURA, SENA ŞÜKRAN; AÇIK, MEHMET EREN; Şentürk, Evren

---

VERSION 1 - REVIEW

**Reviewer**	**1**
**Name**	**Shen, Minxue**
**Affiliation**	**Xiangya Hospital Central South University, Dermatology**
**Date**	**25-Aug-2026**
**COI**	**None**

1.The abstract (and the Strengths and limitations section) explicitly lists local tissue perfusion as a measured parameter, eg. 'non-invasive measurements of ... skin pH, skin temperature, and local tissue perfusion'. However, the methods and primary outcomes include only TEWL, SCH, skin pH, and local skin temperature; no perfusion measurement device or outcome is described anywhere.

2. Two concerns on sample size estimation: 1) it is not stated which specific outcome/parameter the effect size of 0.8 corresponds to; and 2) the study is a multi-parameter, multi-time-point longitudinal design (mixed-effects models) for PI development, and a single two-sample t-test sample-size calculation cannot support the events-per-variable requirements of multivariable Cox models.

3. PI is only detected at discrete time points, so the event time is interval-censored. Moreover, the biophysical parameters are time-varying exposures and covariates. There is also a competing-risk issue, eg. death or ICU discharge before the onset of PI.

VERSION 1 - AUTHOR RESPONSE

Reviewer 1 – Dr. Minxue Shen

Comment1:

The abstract (and the Strengths and limitations section) explicitly lists local tissue perfusion as a measured parameter, eg. "non-invasive measurements of ... skin pH, skin temperature, and local tissue perfusion". However, the methods and primary outcomes include only TEWL, SCH, skin pH, and local skin temperature; no perfusion measurement device or outcome is described anywhere.

Response:

Thank you for this important observation. The reference to local tissue perfusion was intended as general contextual information and was inadvertently included among the study measurements. Local tissue perfusion is not assessed in the SKIN-BAR study. We have therefore removed this term from the Abstract and the Strengths and limitations section to ensure consistency with the Methods and study outcomes.

Comment 2:

Two concerns on sample size estimation: 1) it is not stated which specific outcome/parameter the effect size of 0.8 corresponds to; and 2) the study is a multi-parameter, multi-time-point longitudinal design (mixed-effects models) for PI development, and a single two-sample t-test sample-size calculation cannot support the events-per-variable requirements of multivariable Cox models.

Response:

Thank you for highlighting these important issues. We agree that the outcome underlying the original effect-size assumption was insufficiently specified and that the sample-size rationale required greater clarity. We have therefore revised the sample-size calculation using sacral stratum corneum hydration (SCH) as the primary outcome for the calculation. The calculation was based on previously reported sacral SCH values in patients who developed pressure injuries and those who did not (17.7±3.78 AU vs 20.0±3.92 AU, respectively), corresponding to an effect size of approximately Cohen's d=0.59.

Using a two-sided α of 0.05, 90% statistical power, and an anticipated allocation ratio of 1:2 between patients who develop and do not develop a pressure injury, the minimum required sample size was recalculated as 138 participants (46 and 92 participants, respectively). Allowing for approximately 20% attrition, the target sample size has consequently been revised to 165 participants.

We have revised the Sample size section of the manuscript accordingly and removed the previous calculation based on an unspecified effect size of 0.8. In addition, as detailed in response to Comment 3, the time-to-event analysis plan has been revised to more appropriately reflect the longitudinal structure of the study and the nature of PI ascertainment.

Comment 3:

PI is only detected at discrete time points, so the event time is interval-censored. Moreover, the biophysical parameters are time-varying exposures and covariates. There is also a competing-risk issue, eg. death or ICU discharge before the onset of PI.

Response:

Thank you for this important methodological observation. We agree that the original statistical analysis plan did not adequately account for the interval-censored nature of PI onset, the time-varying nature of the repeated biophysical measurements, or competing events occurring before PI development.

We have therefore revised the statistical analysis plan. The association between longitudinal skin barrier function parameters and incident PI development will be evaluated within a discrete-time survival framework, with follow-up divided according to the predefined skin-assessment intervals. This approach allows PI onset to be represented by the interval in which the injury is first detected rather than assigning an exact event time.

Repeated measurements of TEWL, SCH, skin pH, and local skin temperature will be incorporated as time-varying covariates using the measurements corresponding to each assessment interval. In addition, death and ICU discharge occurring before PI development will be considered competing events in the time-to-event analyses. The multivariable analyses will be restricted to a limited number of prespecified clinically relevant covariates, taking into account the number of observed PI events to minimise model overfitting.

The Statistical analysis section has been revised accordingly to explicitly describe these analytical considerations.

In addition to the revisions made in response to the Editor and Reviewer, we have made a minor editorial change to the manuscript title, replacing "The SKIN-BAR Study" with "The SKIN-BAR Project" to avoid repetition of the word "study". This change does not affect the study design or scope.

We again thank the Editor and Reviewer for their constructive comments, which have helped us improve the methodological clarity and consistency of the study protocol.
